# Assessment of diversity and genetic relationships of *Neonectria ditissima*: the causal agent of fruit tree canker

**DOI:** 10.1186/s41065-016-0011-3

**Published:** 2016-07-01

**Authors:** Marjan Ghasemkhani, Larisa Garkava-Gustavsson, Erland Liljeroth, Hilde Nybom

**Affiliations:** 1 Department of Plant Breeding, Swedish University of Agricultural Sciences, Box 101, 230 53 Alnarp, Sweden; 2 Department of Plant Protection Biology, Swedish University of Agricultural Sciences, Box 102, 230 53 Alnarp, Sweden; 3 Department of Plant Breeding, Swedish University of Agricultural Sciences, Balsgård, Fjälkestadsvägen 459, 29194 Kristianstad, Sweden

**Keywords:** AFLP, Apple canker, Genetic variability, Heterothallism, Mating system, SSR

## Abstract

**Background:**

*Neonectria ditissima* is one of the most important fungal pathogens of apple trees, where it causes fruit tree canker. Information about the amount and partitioning of genetic variation of this fungus could be helpful for improving orchard management strategies and for breeding apple cultivars with high levels of genetically determined resistance. In this study single-spore *Neonectria* isolates originating from both the same and from different perithecia, apple cultivars and apple orchards in Sweden and Belgium, were evaluated for AFLP- and SSR-based genetic similarity and for mating system.

**Results:**

Seven SSR loci produced a total of 31 alleles with an average of 4 alleles per locus, while 11 AFLP primer combinations produced an average of 35 fragments per primer combination and 71 % polymorphic fragments. An AFLP-based analysis of molecular variance (AMOVA) revealed that 89 % of the variation was found within orchards and 11 % between orchards. Genetic similarity among the studied isolates was illustrated with a principal coordinate analyseis (PCoA) and a dendrogram. AFLP-based Jaccard’s similarity coefficients were the highest when single-ascospore isolates obtained from the same perithecium were compared, medium-high for isolates from different perithecia on the same tree, and lowest when isolates from different trees were compared.

**Conclusions:**

Based on the results of PCoA and AMOVA analysis, isolates from the same or geographically close orchards did not group together. Since AFLP profiles differed also when single-ascospore isolates from the same perithecium were compared, the mating system of *N. ditissima* is most likely heterothallic.

## Background

The haploid fungus *Neonectria ditissima* (Tul. & C. Tul.) Samuels & Rossman*,* previously known as *Neonectria galligena* (Bres.) Rossman & Samuels*,* causes cankers on a wide range of trees and shrubs including apple (*Malus*) and pear (*Pyrus*). This pathogen can damage the woody tissue of infected trees substantially, and render fruit production unprofitable in certain areas [5]. Apple canker is mainly associated with mild and wet conditions, and climate therefore has an important impact on the geographic distribution of the pathogen [6]. This pathogen produces both conidia and ascospores under favorable conditions over prolonged periods [20, 33]. The spores usually enter the trees through natural and artificial wound sites e.g., pruning wounds, leaf scars, fruit scars due to chemical thinning or natural abscission, twig stubs, in the crotches of limbs, and even through lesions caused by apple scab. The spores can also be dispersed within and between orchards with infected equipment used by growers [20]. Since the pathogen can cause systemic infections that spread inside the plant, the use of infected rootstocks can probably lead to canker symptoms also in the grafted part (i.e. the proper cultivar) of young apple trees.

In spite of the economic impacts of this pathogen, very little is known about the genetics of the causal agent. Most studies of *Neonectria* have focused on analyses of material from North America. In a study of Mahoney et al. [17], Restriction Fragment Length Polymorphism (RFLP) of ribosomal, mitochondrial, and anonymous nuclear DNA was used to determine the origin of *N. coccinea* var. *faginata* in North America, and also to estimate haplotype and nucleotide diversity in this species as well as in *N. ditissima.* Plante et al. [25] investigated genetic diversity of *N. ditissima* and *N. coccinea* var. *faginata* using Random Amplified Polymorphic DNA (RAPD) and ribosomal DNA polymorphisms, and found that diversity of *N. ditissima* on various hardwood hosts in North America was higher than that of *N. faginata* (*Nectria coccinea var. faginata*). Based on a combined analysis of elongation factor 1-alpha (EF1-α), RNA polymerase II second largest subunit (RPB2), and β-tubulin gene regions, Castlebury et al. [4] report that *N. ditissima* appears to consist of two major groups; one with primarily North American isolates, and another with European isolates from *Fagus*, *Malus* and *Salix*.

Fungi can be self-fertile and undergo sexual reproduction (haploid selfing) on a single colony or be self-sterile and require two colonies of different mating types for sexual reproduction (out-crossing) [2]. However, many fungi can revert to haploid selfing if a suitable partner is not available.

Studies of fungal population biology can assist in understanding the evolution of disease in agriculture and how it is affected by, e.g., the life cycle of the pathogen [26]. Population genetic analyses can also provide information about the amount of sexual recombination in fungal populations [32]. Sexual pathogen populations can produce new combinations of alleles including fungicide resistance alleles, which allow the pathogen to rapidly adapt to changes in the environment [21]. Analysis of sibling ascospore offspring can be used to more closely define the nature of mating systems [7]. Out-crossing was thus concluded when RAPD- and AFLP-markers detected genetic variation among sibling single-ascospore progeny from apothecia of *Cladonia xoerkeana*, *C. galindezii*, and *C. portentosa* [27]. However, previously collected data for *N. ditissima* are conflicting; based on the morphology of macroconidia and ascospores, El-Gholl et al. [9] reported this species to be homothallic while out-crossing was reported in an older study of ascospore morphology [14].

## Methods

### Fungal isolates

**Fig. 1:**
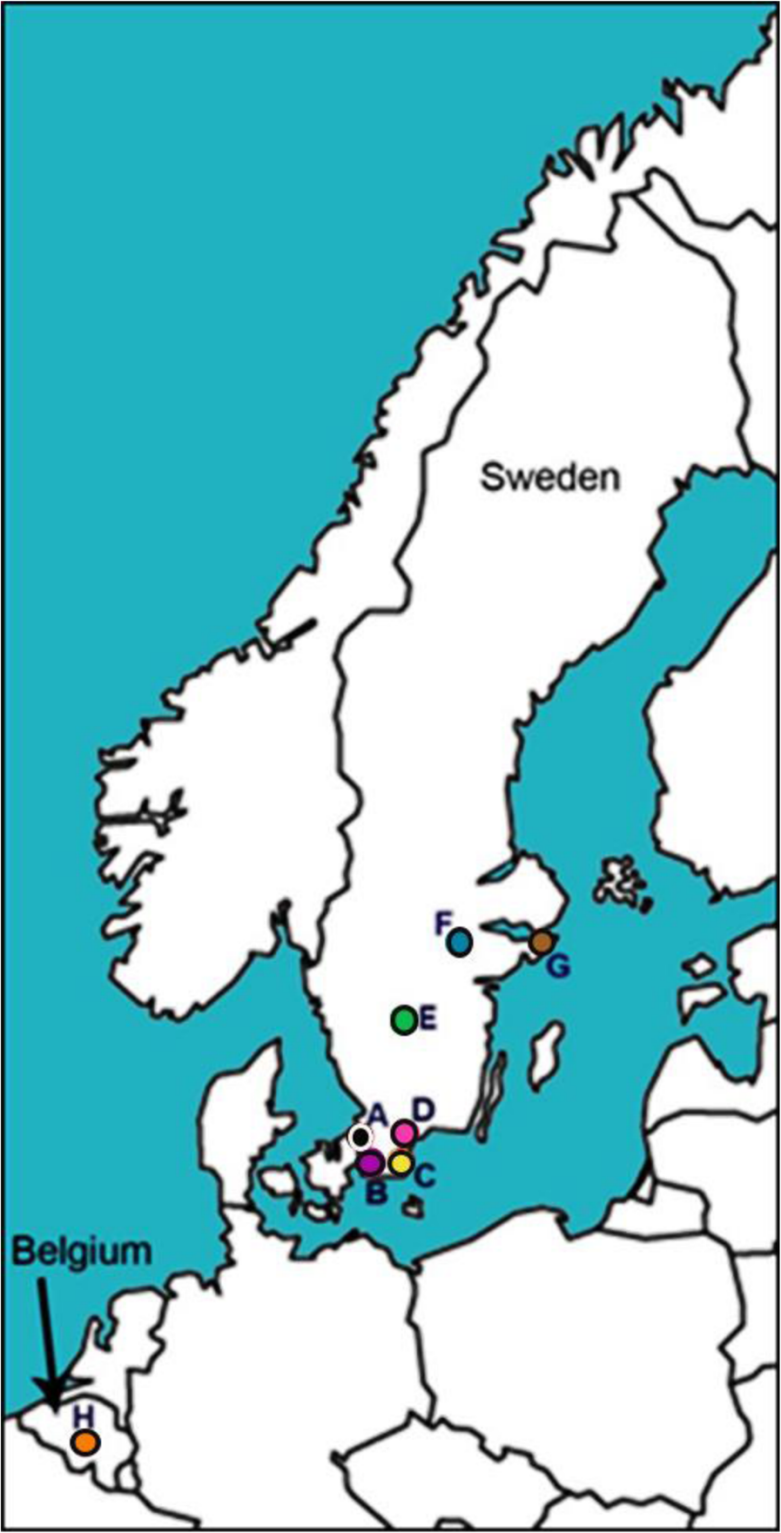
Map showing origin of isolates in Sweden and Belgium, A (black circle): Jonstorp, B (purple circle): Bjärred, C (yellow circle): Kivik, D (pink circle): Balsgård, E (green circle): Jönköping, F (blue circle): Julita, G (brown circle): Stockholm, H (orange circle): Gembloux

Ascospore samples were collected from vegetatively propagated apple cultivars grown in seven different apple orchards in southern Sweden and from one orchard in Belgium (Fig. 1 and Table 1). In each orchard, 2–10 trees, located in different places in the orchard, with visible canker lesions were chosen, and pieces of bark and wood bearing perithecia were collected. Individual perithecia were surface-sterilized by immersion for 30 s in 50 % ethanol and for 30 s in 1.5 % sodium hypochloride, and then washed in sterile distilled water. Each perithecium was crushed in 100 μl of sterile distilled water. About 50 μl of the spore suspension was plated on agar (Difco) plates containing tetracycline (100 ppm) and incubated at 21 °C. One day later, 1–3 germinated ascospores were recovered individually using a tiny needle under a stereo-microscope, and transferred onto fresh 2 % malt extract agar (MEA, Merck) plates and left to grow for 14–18 days to produce single-ascospore cultures [1]. Correct species determination was ascertained by observation of colony morphology and by PCR amplification of DNA samples using species-specific primers previously developed for *N. ditissima* [13].

**Table 1:** Geographic origin of the 44 isolates of *N. ditissima* used in this study. Number 1–38 were isolated in 2013 and the remainder in 2014

Number	CBS	Apple cultivar	Origin
*Isolate 1*	139272	Aroma	Jonstorp, Sweden (N 56°13', E 12°40')
*Isolate 2*	139273	Ingrid Marie	Jonstorp, Sweden
*Isolate 3*	139271	Rubinstar	Jonstorp, Sweden
*Isolate 4*	139269	Åkerö	Julita, Sweden (N 59°11', E 16°1')
*Isolate 5*	139270	Norrstack	Julita, Sweden
*Isolate 6*	139388	Oranie	Jönköping, Sweden (N 57°46' N, E 14°9')
*Isolate 7*	139268	James Grieve	Jönköping, Sweden
*Isolate 8*	139390	Discovery	Kivik, Sweden (N 55°41', E 14°13')
*Isolate 9*	139389	Ingrid Marie	Kivik, Sweden
*Isolate 10*	139265	John Standish	Balsgård, Sweden (N 56°6', E 14°9')
*Isolate 11*	139267	Brite Spur	Balsgård, Sweden
*Isolate 12*	139327	Pigeon	Balsgård, Sweden
*Isolate 13*	139266	McIntosh	Balsgård, Sweden
*Isolate 14*	139255	Tompkin’s King	Balsgård, Sweden
*Isolate 15- RP1A1*	139260	Red Delicious	Balsgård, Sweden
*Isolate 16- RP2A1*	---------	Red Delicious	Balsgård, Sweden
*Isolate 17- RP3A1*	---------	Red Delicious	Balsgård, Sweden
*Isolate 18- RP3A2*	---------	Red Delicious	Balsgård, Sweden
*Isolate 19- RP3A3*	---------	Red Delicious	Balsgård, Sweden
*Isolate 20*	139262	Beacon	Balsgård, Sweden
*Isolate 21- HP1A1*	139263	Holsteiner Cox	Balsgård, Sweden
*Isolate 22- HP1A2*	---------	Holsteiner Cox	Balsgård, Sweden
*Isolate 23- HP1A3*	---------	Holsteiner Cox	Balsgård, Sweden
*Isolate 24- HP3A1*	---------	Holsteiner Cox	Balsgård, Sweden
*Isolate 25- HP3A2*	---------	Holsteiner Cox	Balsgård, Sweden
*Isolate 26- HP2A1*	---------	Holsteiner Cox	Balsgård, Sweden
*Isolate 27- HP2A2*	---------	Holsteiner Cox	Balsgård, Sweden
*Isolate 28- HP2A3*	---------	Holsteiner Cox	Balsgård, Sweden
*Isolate 29*	139264	Freiherr von Berlepsch	Balsgård, Sweden
*Isolate 30- GP1A1*	139261	Gravensteiner	Balsgård, Sweden
*Isolate 31- GP1A2*	---------	Gravensteiner	Balsgård, Sweden
*Isolate 32- GP1A3*	---------	Gravensteiner	Balsgård, Sweden
*Isolate 33- GP2A1*	---------	Gravensteiner	Balsgård, Sweden
*Isolate 34- GP2A2*	---------	Gravensteiner	Balsgård, Sweden
*Isolate 35- GP2A3*	---------	Gravensteiner	Balsgård, Sweden
*Isolate 36*	139275	Gloster	Bjärred, Sweden (N 55°43', E 13°1')
*Isolate 37*	139274	Ingrid Marie	Bjärred, Sweden
*Isolate 38*	139503	Elise	Bjärred, Sweden
*Isolate 39*	139259	Discovery	Gembloux, Belgium N 54°34', E 4°42')
*Isolate 40*	139257	Api Etoile	Gembloux, Belgium
*Isolate 41*	139256	Baron	Gembloux, Belgium
*Isolate 42*	139258	Reinette	Gembloux, Belgium
*Isolate 43*	139277	Eva-Lotta	Stockholm, Sweden (N 59°19', E 18°4')
*Isolate 44*	139276	Södermanlandsäpple	Stockholm, Sweden

Isolator: Marjan Ghasemkhani

CBS-KNAW Fungal Biodiversity Center, Utrecht, The Netherlands

R: Red Delicious, G: Gravensteiner, H: Holsteiner Cox, P1-3: different perithecia on the same tree, A1-3: different single-ascospore isolates from the same perithecium

### DNA extraction

For DNA extraction of the isolates of *N. ditissima*, an agar plug of each fungal culture was transferred into liquid medium, malt extract/glucose/peptone (MGP, 20 g malt extract, 20 g glucose and 1 g peptone in 1 L water at pH 5.5) [28], and grown at room temperature on a shaker platform for 10 days. The mycelium was harvested by filtration through Whatman No. 1 filter paper, washed two times with distilled water, freeze-dried and stored at −20 °C until processing. DNA was extracted with DNeasy Plant Mini Kit (Qiagen GmbH, Hilden, Germany) according to manufacturer’s instructions. DNA quantification was performed using electrophoresis in 1.5 % agarose gel containing ethidium bromide and visualized by UV fluorescence, and concentrations and purity (260/280 nm > 1.8) were estimated using a ND-1000 spectrophotometer (NanoDrop, Wilmington, USA).

### Amplification with SSR markers

Seven primer pairs, previously developed from isolates of *N. ditissima* sampled on black birch, *Betula nigra* [19], were used for SSR analysis. Polymerase chain reaction (PCR) was performed in a total volume of 25 μL in 2.5 μl of 10X PCR buffer, 0.2 mM dNTP mix, 0.5 μM of each primer, 1.5 mM MgCl_2_, 1 Unit of *Taq* DNA polymerase (Thermo Scientific, San Jose, USA) and 10 ng genomic DNA. Amplifications were performed in a S1000 thermal cycler (BIO-RAD, San Francisco, USA) with the following cycling profile: 94 °C for 5 min, 10 cycles of 94 °C for 30 s, 58 °C for 45 s, and 72 °C for 45 s, followed by 30 cycles of 94 °C for 30 s, 53 °C for 45 s, and 72 °C for 45 s, a final extension step for 7 min at 72 °C.

### Amplification with AFLP markers

AFLP analysis of DNA was carried out with the AFLP Microbial Fingerprinting kit (Applied Biosystems, Foster City, CA, USA) according to the recommendations of the manufacturer. Restriction-ligation reaction and preselective amplification of the AFLP procedure were carried out using the AFLP® Ligation and Preselective Amplification Module of the Microbial Fingerprinting kit. Briefly, in the restriction-ligation reactions 10 ng of genomic DNA was simultaneously digested with *Eco*RI and *Mse*I endonucleases (New England Biolabs, Beverly, Mass., USA) and ligated to *Eco*RI and *Mse*I adaptors in a final volume of 11 μl containing 10X T4 DNA ligase buffer with ATP, *Mse*I, *Eco*RI, T4 DNA ligase, BSA (all: New England Biolabs, Mass., USA), 0.5 M NaCl, and *Eco*RI-adaptors and *Mse*I-adaptors (Applied Biosystems). Restriction-ligation reactions were carried out for 2 h at 37 °C in a Bio-Rad S1000 thermal cycler. After dilution of DNA with TE_0.1_ buffer (20 mM Tris–HCl, 0.1 mM EDTA, pH 8.0), for the preselective amplification, 4 μl diluted DNA was amplified with the *Eco*RI and *Mse*I core primer sequences (Applied Biosystems) in a final volume of 20 μl. Selective amplification was performed with 11 primer combinations of fluorescently labelled primers binding to the *Eco*RI-adaptor and unlabeled primers binding to the *Mse*I-adaptor: E+AA/M+CA, E+A/M+CT, E+T/M+CT, E+AC/M+CT, E+AC/M+CA, E+AG/M+CT, E+AT/M+CT, E+AA/M+CT, E+AG/M+CG, E+AC/M+CC, E+AC/M+CG, by using 3 μl of the diluted preselective amplification reaction mixture in a final volume of 20 μl. All PCR amplifications were carried out in a BioRad S1000 thermal cycler.

In order to check the reproducibility of AFLP amplification, 5–8 randomly selected samples were added as technical replicates to each 96-well plate before running the PCR.

### Fragment detection and analysis

The reaction products were mixed with 0.1 x TE buffer and a ROX™ dye-labeled size standard with fragments in the range 58–362 bp for SSR and 50–500 bp for AFLP markers and electrophoresed on an ABI 3130xl DNA analyzer (Applied Biosystems) using a 36 cm capillary array and POP-7™ polymer. Trace files were analyzed with GeneMarker® software (Version 1.85, SoftGenetics LLC, State College, PA, USA). AFLP markers producing weak or ambiguous signals, relative fluorescence unit (RFU) >100, were not considered.

### Data analysis

Distinct and well-resolved SSR and AFLP fragments were scored using the GeneMarker® program, and converted to binary data (one separate file for SSR data and one for AFLP data) based on the presence or absence of the discriminatory bands (1 for presence and 0 for absence). Genetic diversity was evaluated using the program POPGENE v 1.32 [36], and the following parameters: number of alleles (NA, for SSR), number of polymorphic loci (NPL, for AFLP), percentage of polymorphic loci (PPL, for AFLP), Nei’s gene diversity (H, both marker types), and Shannon’s information index (I, both marker types). Pair-wise comparisons of single-ascospore isolates were conducted using Jaccard’s similarity coefficient (SJ), according to the formula *SJ* = (*N*_*ab*_)/(*N*_*a*_ + *N*_*b*_ − *N*_*ab*_), where N_ab_ is number of shared bands; N_a_ and N_b_ are number of bands in isolates a and b, respectively [34]. Genetic relationships among the studied single-ascospore isolates were also assessed by two multivariate principal coordinate analyses (PCoA) performed with NTSYS 2.02 (Applied Biostatistics, Setauket, NY, USA) for SSR and AFLP data, respectively. A dendrogram was constructed by binary band matching using the unweighted pairwise group method with arithmetic mean (UPGMA) with NTSYS 2.02 [30] for single ascospore isolates sampled from perithecia on three trees at Balsgård and investigated with AFLP. A cophenetic correlation coefficient was calculated using a Mantel test [18] in order to check the goodness-of-fit of the cluster analysis with the matrix on which it was based. A Mantel test was also performed to investigate possible correlation between two genetic distance matrices based on SSR and AFLP markers using NTSYS 2.02. An analysis of molecular variation (AMOVA) was calculated for partitioning of AFLP marker variation between and within orchards, using Arlequin 3.11 [10].

## Results and discussion

### Evaluation of primers used

Seven different SSR primer pairs were used (Table 2), with number of alleles varying from 2 to 7 per locus. Since all single-ascospore isolates collected from a single tree produced identical profiles, only one isolate for each tree (a total of 28) was used in subsequent analyses. Shannon’s information index varied from 0.15 for primer NdGGT39 to 0.51 for primer NdGGT3. NdGGT39 also yielded the highest Nei’s gene diversity (0.69) while NdGGT23 produced the lowest (0.13). The average gene diversity for the seven primer pairs was 0.28. When evaluated on *N. ditissima* isolates collected from a single black birch population, gene diversity obtained with the same seven primer pairs was considerably higher, ranging from 0.11 to 0.86, and with an average of 0.48 [19]. These results suggest that the fungal populations occurring on apple were less variable than the single population occurring on black birch.

**Table 2:** Number of alleles (NA), Nei’s gene diversity (H) and Shannon’s information index (I) for seven SSR primer pairs used to study variation among 28 isolates of *N. ditissima*

Primer name	NA	H	I
NdCAA3	7	0.24	0.38
NdCAA11	7	0.17	0.30
NdGGT2	5	0.22	0.36
NdGGT3	3	0.35	0.51
NdGGT23	2	0.13	0.26
NdGGT39	5	0.69	0.15
NdGGT44	2	0.14	0.26
Total	31		
Average	4	0.28	0.32

**Fig. 2:**
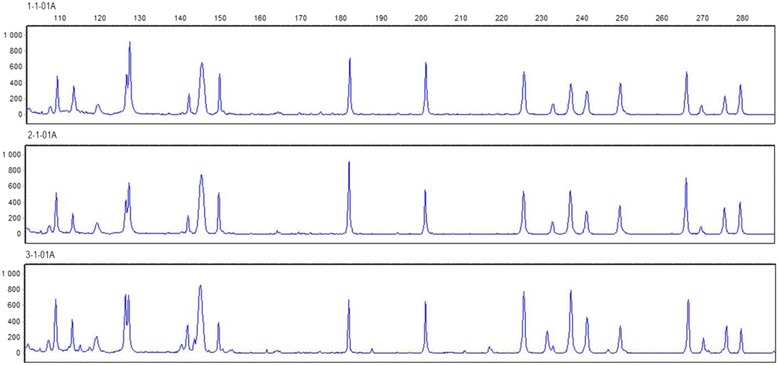
Electropherograms generated from 3 runs on an ABI 3130xl of the same sample that obtained with the primer combination E+T/M+CT. They indicated high reproducibility of AFLP marker

Eleven different AFLP primer combinations (Table 3) were used in this study to generate AFLP profiles for 44 isolates. A total of 391 fragments (ranging from 50 to 500 bp) were identified, of which 272 were polymorphic with reliable and clear reading. The lowest number of fragments was detected for the combination E+AC/M+CG and the highest for the combination E+T/M+CT, with 21 and 58 fragments, respectively. The percentage of polymorphism ranged from 55 % (in the combination E+A/M+CT) to 96 % (in the combination E+AC/M+CC). The combination E+AC/M+CC also produced the highest values for Nei’s gene diversity and Shannon’s information index while the combination E+AA/M+CT produced the lowest values. Evaluation of the technical replicates, which were included in each 96-well plate, demonstrated high reproducibility of produced band patterns (Fig. 2).

**Table 3:** Number of loci (NL), number of polymorphic loci (NPL), percentage of polymorphic loci (PPL), Nei’s gene diversity (H) and Shannon’s information index (I) for 11 AFLP primer combinations used to study variation among 44 isolates of *N. ditissima*

Primer combinations	NL	NPL	PPL	H	I
E+AA/M+CA	40	35	87	0.20	0.33
E+A/M+CT	56	31	55	0.10	0.17
E+T/M+CT	58	37	64	0.14	0.22
E+AC/M+CT	29	18	62	0.18	0.28
E+AC/M+CA	25	18	72	0.14	0.23
E+AG/M+CT	33	25	76	0.17	0.26
E+AT/M+CT	38	27	71	0.13	0.21
E+AA/M+CT	42	25	59	0.09	0.16
E+AG/M+CG	22	16	73	0.17	0.27
E+AC/M+CC	27	26	96	0.21	0.34
E+AC/M+CG	21	14	67	0.13	0.22
Total	391	272			
Average	35	25	71	0.15	0.24

Overall, SSR markers showed a higher level of polymorphism (100 %) than the AFLP markers (71 %). These findings are consistent with other studies comparing the level of polymorphism detected with SSR and AFLP markers [11, 16], and can be explained by the fact that SSR markers detect multiple alleles at a given locus, usually pre-selected for its high variability, whereas AFLP assays mainly detect single alleles at multiple loci randomly distributed in the genome.

A Mantel test of the two genetic distance matrices derived from SSR and AFLP data, respectively, yielded a correlation coefficient of only 0.12 (*P* = 0.89), which indicates that the two marker systems have not produced concordant results. Genetic distances are often closely correlated when different multi-locus marker methods (like AFLP, RAPD and ISSR) are compared [34]. By contrast, comparison of a multi-locus method with SSR markers can result in poor correlation, especially when number of SSR bands evaluated is too low. The present study is a first report to assess genetic variation among and within populations of *N. ditissima* in European countries using both SSR and AFLP markers.

### Genetic variation between and within orchards

Analysis of molecular variation (AMOVA) among 44 AFLP-screened isolates, obtained from eight different orchards, revealed only 11 % genetic differentiation among orchards, while the remaining 89 % of the variation resided within orchards. As expected, number of polymorphic bands detected in each orchard was closely associated with the number of isolates investigated. The highest number of polymorphic AFLP bands was observed in the orchard at Balsgård (201 bands among 26 isolates from 10 trees) followed by Gembloux (102 bands among four isolates) while Jönköping had the lowest number (42 bands among 2 isolates). The highest number of SSR bands was also found at Balsgård (14) and Gembloux (12) while the lowest number (3) was identified in Stockholm (two isolates sampled) (Table 4).

**Table 4:** Number of polymorphic loci (NPL) and percentage of polymorphic loci (PPL) for AFLP, number of polymorphic alleles (NPA) and percentage of polymorphic alleles (PPA) for SSR, Nei’s gene diversity (H) and Shannon’s information index (I) for populations of *Neonectria ditissima* from different orchards based on AFLP with 44 isolates and SSR markers with 28 isolates

		NPL	PPL	H	I
AFLP Marker					
*Genetic diversity of the eight populations*					
Orchards	Balsgård, Sweden	201	51	0.13	0.21
	Bjärred, Sweden	88	22	0.10	0.14
	Kivik, Sweden	64	16	0.08	0.11
	Julita, Sweden	60	15	0.07	0.10
	Jonstorp, Sweden	80	20	0.09	0.13
	Jönköping, Sweden	42	11	0.05	0.07
	Stockholm, Sweden	67	17	0.08	0.12
	Gembloux, Belgium	102	26	0.10	0.15
	Mean	88	22.2	0.09	0.13
*Genetic diversity among 44 isolates*					
44 isolates		272	69	0.14	0.23
		NPA	PPA	H	I
SSR Marker					
*Genetic diversity of the eight populations*					
Orchards	Balsgård, Sweden	14	45	0.14	0.22
	Bjärred, Sweden	9	29	0.13	0.18
	Kivik, Sweden	7	23	0.11	0.16
	Julita, Sweden	5	16	0.08	0.11
	Jonstorp, Sweden	6	19	0.09	0.12
	Jönköping, Sweden	6	19	0.10	0.13
	Stockholm, Sweden	3	10	0.05	0.07
	Gembloux, Belgium	12	39	0.15	0.22
	Mean	7.7	25	0.11	0.15
*Genetic diversity among 28 isolates*					
28 isolates		31	100	0.19	0.32

When calculated at the species level, Nei’s gene diversity and Shannon’s information index were 0.14 and 0.23 based on AFLP data (44 isolates in total), respectively, and 0.19 and 0.32 based on SSR data (28 isolates), respectively (Table 4). At the population (i.e. orchard) level, Balsgård and Gembloux had the highest values for Nei’s gene diversity and Shannon’s information index for both AFLP and SSR markers. When based on AFLP data, Nei’s gene diversity and Shannon’s information index was 0.13 and 0.21, respectively, at Balsgård, and 0.05 and 0.07, respectively, at Jönköping where the lowest values were encountered. For SSR marker data, Gembloux and Balsgård had a Nei’s gene diversity of 0.15 and 0.14, respectively, and a Shannon’s information index of 0.22 for both orchards. The lowest values were instead encountered at Stockholm (0.05 and 0.07, respectively). It should, however, be noted that all the estimates of intra-population variation are based on very few isolates and are therefore unlikely to be representative of the species at large. In the previous study where these SSR primer pairs were developed and evaluated on a single *N. ditissima* population sampled on black birch, Nei’s gene diversity was considerably higher (0.48) [19]. The latter population was, however, also much larger (38 isolates) which may explain the difference in diversity.

Only 11 % of the variation resided between populations in the present study as assessed by the AFLP markers. Rather low values for population differentiation have been reported also in other studies on molecular variation in fungi. AFLP-based studies have thus shown that 7 % of the total genetic variability occurred between populations of *Fusarium pseudograminearum* [12] and 14 % between populations of *Phytophthora colocasiae* [24] while 11 % of SNP-based variability occurred between populations of *Ustilaginoidea virens* [29]. Low genetic differentiation is often hypothesized to result from high levels of gene flow thus preventing geographic subdivision [15]. Since the apple canker fungus occurs on cultivated crops, gene flow is probably augmented by movement of plant material from one area to another, e.g. from commercial plant nurseries in Belgium to orchards in Sweden as well as between orchards. Another possible explanation for the lack of strong inter-populational differentiation among isolates is a high degree of spontaneous mutations. A large number of spores is produced by the fungus in a short period of time, and the diversity could therefore be influenced by mutations [3].

### Mating system of *N. ditissima*

In order to determine the most likely mode of reproductive system in *N. ditissima* and to quantify genetic variation among isolates at different levels of genetic relatedness, single-ascospore isolates derived from the same or from different perithecia on a single tree, were analyzed with both AFLP and SSR markers (Table 1). A set of 45 single-ascospore isolates representing five different trees from different orchards (nine single-ascospore isolates from three perithecia on each tree) was analyzed with SSR, yielding identical profiles when isolates from the same tree were compared (data not shown).

Subsequently, a set of 19 single-ascospore isolates sampled in the orchard at Balsgård (five from three perithecia on a ‘Red Delicious’ tree, eight from three perithecia on a ‘Holsteiner Cox’ tree and six from two perithecia on a ‘Gravensteiner’ tree) was analyzed with AFLP. Average pairwise similarity was analyzed at three levels; 1) among different single-ascospore isolates derived from the same perithecium, b) among single-ascospore isolates derived from different perithecia on the same tree, and c) among single-ascospore isolates derived from different trees (Table 5). For level 1, pairwise comparisons of isolates using Jaccard's similarity coefficient ranged from 0.73 to 1.00 with a mean of 0.85. For level 2, Jaccard's similarity coefficients ranged from 0.60 to 0.87 with a mean of 0.76, suggesting that genetic relatedness is lower among isolates from different perithecia compared to isolates originating from the same perithecium. Finally, for level 3, Jaccard's similarity coefficients calculated on comparisons between isolates from different trees in the same orchard ranged from 0.44 to 0.70, with a mean of 0.56.

**Table 5:** AFLP-based variation estimated with Jaccard’s similarity coefficients among single-ascospore isolates originating from within the same or from different perithecia of *N. ditissima*

Within the same perithecium		Between different perithecia on the same tree		Between different perithecia on different trees in the same orchard	
RP3 :		RP1×RP2×RP3:		RP×GP:	
Mean	0.96	Mean	0.75	Mean	0.56
Max	1	Max	0.85	Max	0.68
Min	0.94	Min	0.60	Min	0.45
GP1:		GP1×GP2:		RP×HP:	
Mean	0.76	Mean	0.77	Mean	0.56
Max	0.84	Max	0.87	Max	0.69
Min	0.73	Min	0.61	Min	0.44
GP2 :		HP1×HP2×HP3:		GP×HP:	
Mean	0.85	Mean	0.76	Mean	0.58
Max	0.90	Max	0.84	Max	0.70
Min	0.80	Min	0.63	Min	0.45
HP1:		Mean	**0.76**	Mean	**0.56**
Mean	0.86				
Max	0.90				
Min	0.84				
HP2:					
Mean	0.86				
Max	0.90				
Min	0.83				
Mean	**0.85**				

R, G and H are different apple cultivars; R: ‘Red Delicious’, G: ‘Gravensteiner’, H: ‘Holsteiner Cox’, P1-3: different perithecia of each cultivar

Bold data indicate the total average pairwise similarity at three levels

Since fingerprint profiles evidently vary amongst sibling progeny, outcrossing seems to occur in *N. ditissima.* Still, occasional selfing cannot be ruled out since two of the isolates from perithecium number 3 on ‘Red Delicious’ were highly identical and the third differed only to a very minor extent from the other two (Jaccard's similarity coefficient = 0.94).

Both haploid selfing [9] and outcrossing [14] has previously been reported in *N. ditissima* according to morphological characterization of ascospores. In general, sexual recombination has the potential to increase genotype diversity since novel recombinants are created, and genetic recombination through sexual crosses have been shown to be correlated with genetic diversity [29]. In the current study, genetic variation among single-ascospore isolates from different perithecia was higher than single-ascospore isolates from the same perithecium, which suggests that each tree has been colonized by several genetically different spores. Moreover, perithecia on different trees in the same orchard produced even more genetically different spores. Obviously, colonization of the fungus within an orchard involves several different, sexually derived ascospores. Management of diseases caused by sexually reproducing pathogens becomes more difficult due to the constant appearance of new genotypes, which increase the variation of features like fungicide resistance. In addition, *N. ditissima* produces asexual spores during a large part of the year. Pathogens that reproduce both sexually and asexually are especially difficult to control, since aggressive isolates selected after sexual reproduction can increase asexually and then disperse widely within a short time-span [22].

### Phenetic analyses

**Fig. 3:**
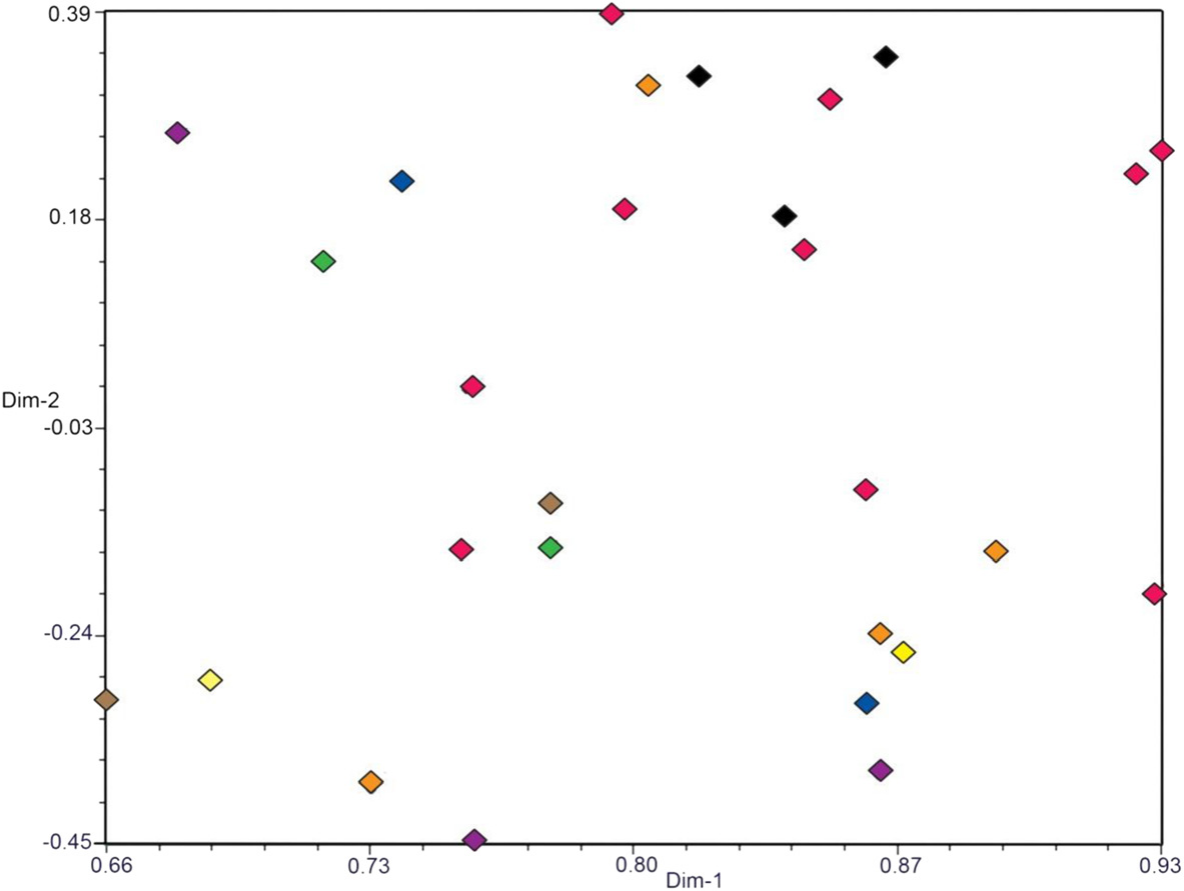
Principal coordinate analysis (PCoA) based on SSR data with 28 isolates. (black diamond): Jonstorp, (blue diamond): Julita, (green diamond): Jönköping, (yellow diamond): Kivik, (pink diamond): Balsgård, (purple diamond): Bjärred, (orange diamond): Gembloux, (brown diamond): Stockholm

**Fig. 4:**
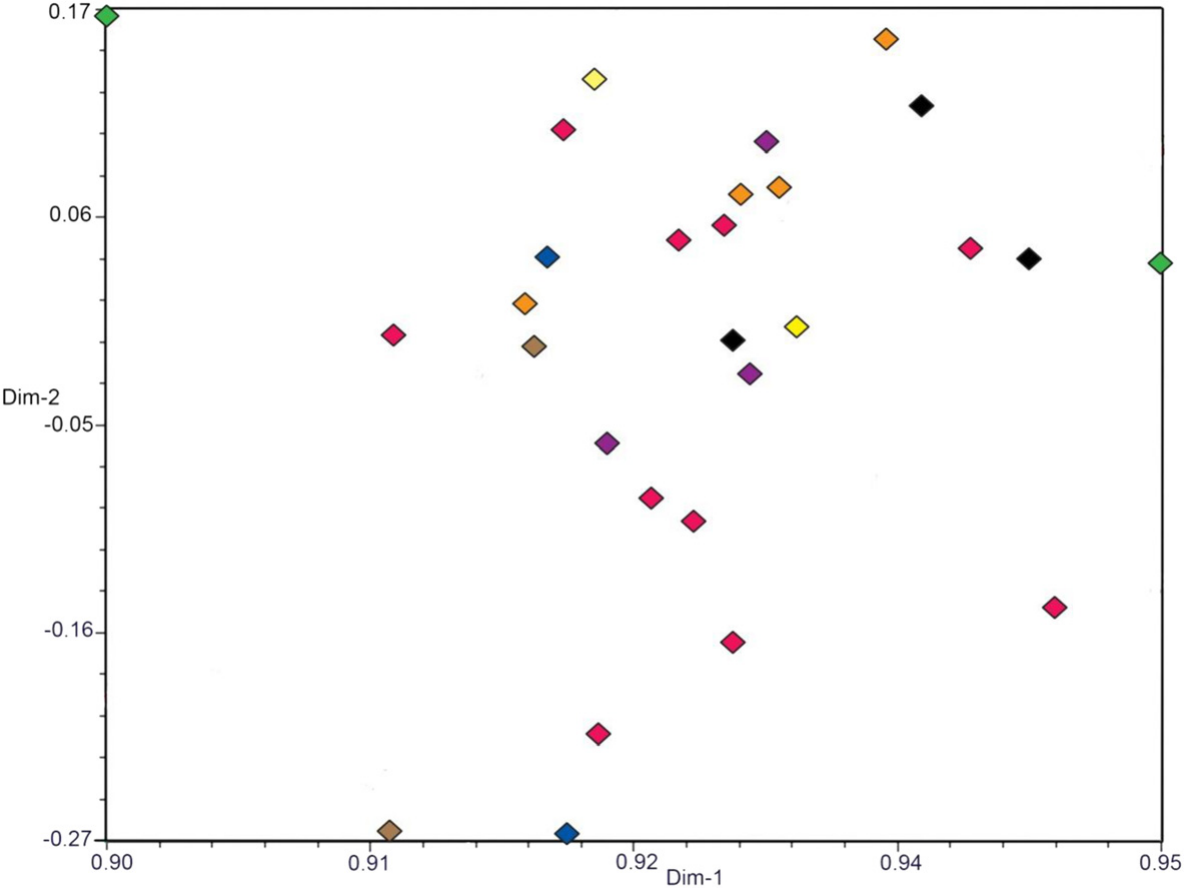
Principal coordinate analysis (PCoA) based on AFLP data with 28 isolates. (black diamond): Jonstorp, (blue diamond): Julita, (green diamond): Jönköping, (yellow diamond): Kivik, (pink diamond): Balsgård, (purple diamond): Bjärred, (orange diamond): Gembloux, (brown diamond): Stockholm

Both SSR and AFLP data were used for estimating the distribution of genetic variation among and within orchards. An SSR-based principal coordinates analysis (PCoA) of similarities among 28 isolates did not produce any distinct groupings of isolates from different trees growing in the same orchard (Fig. 3). Neither was an impact from geographical distances among the eight sampled orchards detected. In this analysis, the first two dimensions (dim-1 and dim-2) explained 65.7 and 6.9 % of the genetic variation, respectively. A PCoA based on AFLP data for the same 28 isolates similarly failed to produce any groupings of the material (Fig. 4), and 85.7 and 1.4 % of the genetic variation were explained by dim-1 and dim-2, respectively. Obviously, isolates from the same or geographically close orchards did not group together in the PCoA analysis with either marker type.

**Fig. 5:**
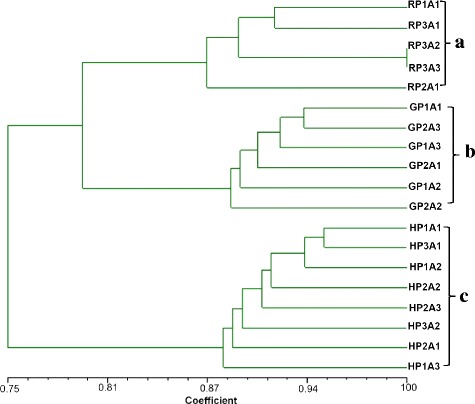
Dendrogram obtained with UPGMA based on AFLP data of single-ascospore isolates arising from different perithecia of *N. ditissima*. R: Red Delicious, G: Gravensteiner, H: Holsteiner Cox, P1-3: different perithecia, A1-3: different single-ascospores. Vertical bars show all isolates arising from different perithecia of **a** ‘Gravensteiner’, **b** ‘Red Delicious’ and **c** ‘Holsteiner Cox’

When the AFLP data for single-ascospore isolates arising from different perithecia on three trees at Balsgård was subjected to a cluster analysis, three distinct groups were formed, each with isolates originating from the same tree, namely groups A (isolates from a ‘Red Delicious’ tree at Balsgård), B (isolates from a ‘Gravensteiner’ tree at Balsgård) and C (isolates from a ‘Holsteiner Cox’ tree at Balsgård) (Fig. 5). The cophenetic correlation coefficient was 0.94, indicating that the dendrogram was a good fit of the original data [18].

The identity of the cultivar from which the perithecia were sampled, did not appear to influence distribution of genetic variation of the resulting isolates. A possible explanation might be that there is random mating in the *N. ditissima* population. By contrast, significant differences among fungal populations of *Venturia inaequalis,* the causal agent of apple scab, collected from different apple cultivars indicated non-random mating [35].

Pathogenicity traits of fungal pathogens can be augmented by so-called pestification processes, which affect mainly growth and dispersal. Previous studies have thus shown that apple domestication has had a considerable impact on the modification of pathogenicity of *V. inaequalis* [31] and that the aggressiveness of this fungus has increased during the domestication process [8]. The extent to which these findings, obtained for the hemibiotrophic, ephemeral (trees are re-colonized each year) and multi-race fungus *Venturia*, can be expected also in a necrotrophic and polyphagous fungus like *Neonectria* is not known. Evolution of fitness-related traits is extremely dependent on the different life histories such as predominance of sexual over asexual reproduction [26].

Information on the distribution of genetic diversity of fungal populations may indicate how spread of the pathogen is accomplished, and this can be used to predict how long a control measure is likely to be effective [23]. A previous study has shown that fungal populations with regular sexual reproduction recombine their genes into new combinations while populations with asexual reproduction have a more limited number of different gene combinations [23]. In the present study, sexually produced spores were used to evaluate genetic variation. The intra-population variation detected is probably largely due to sexual recombination. Gene flow, mutation and selection are hypothesized as the most important mechanisms of variation in *N. ditissima* populations. Strategies for improved control should therefore include restricted movement of infected plant material, or contaminated equipment.

## Conclusions

To the best of our knowledge, studies of genetic variation in *N. ditissima* on *Malus domestica* have not been reported before in Sweden or Belgium. In this study, genetic diversity of *N. ditissima* was estimated at an inter-population level using isolates from different apple orchards in Sweden and from one orchard in Belgium since trees grown in commercial Swedish orchards are often imported from Belgium. In addition, the mating system of the pathogen was investigated at an intra-population level by comparing sibling single-ascospore progeny either from a single perithecium or from different perithecia of the same tree. First, Simple Sequence Repeat (SSR) was used, which is a single-locus method with co-dominantly inherited bands. Each primer pair produces one band in each isolate (the studied species is haploid). These bands can have different size in different isolates. Since total number of polymorphic SSR alleles turned out to be rather low, the Amplified Fragment Length Polymorphism (AFLP) method was applied to complement the SSR data. AFLP is a multi-locus method with dominantly inherited bands, with each primer pair producing multiple bands in each isolate. When several isolates are compared, individual AFLP bands are either present or absent [34].

## Abbreviations

AFLP, amplified fragment length polymorphism; AMOVA, analysis of molecular variance; EF1-α, elongation factor 1-alpha; ISSR, application of inter simple sequence repeat; PCoA, principal coordinates analysis; RAPD, random amplified polymorphic DNA; RPB2, RNA polymerase II second largest subunit; SSR, simple sequence repeat; UPGMA, unweighted pairwise group method with arithmetic mean

## References

[1] Bernier L, Hubbes M (1990). Mutations in *Ophiostoma ulmi* induced by N-methyl-N'-nitro-N-nitroso-guanidine. Can J Bot.

[2] Billiard S, López‐Villavicencio M, Hood M, Giraud T (2012). Sex, outcrossing and mating types: unsolved questions in fungi and beyond. J Evol Biol.

[3] Carlile MJ, Watkinson SC, Gooday GW (2001). The Fungi.

[4] Castlebury LA, Rossman AY, Hyten AS (2006). Phylogenetic relationships of *Neonectria/Cylindrocarpon* on *Fagus* in North America. Can J Bot.

[5] Chatelet DS, Wistrom CM, Purcell AH, Rost TL, Matthews MA (2011). Xylem structure of four grape varieties and 12 alternative hosts to the xylem-limited bacterium *Xylella fastidious*. Ann Bot-London.

[6] CPCI (2014). Crop Protection Compendium on Internet.

[7] Czembor PC, Arseniuk E (2000). Segregation and recombination of PCR based markers in sexual progeny of *Phaeosphaeria* species. Mycol Res.

[8] De Gracia M, Cascales M, Expert P, Bellanger M-N, Le Cam B, Lemaire C (2015). How did host domestication modify life history traits of its pathogens?. PLoS One.

[9] El-Gholl N, Barnard E, Schroeder R (1986). Homothallism in *Nectria galligena*. Can J Bot.

[10] Excoffier L, Laval G, Schneider S (2005). Arlequin (version 3.0): an integrated software package for population genetics data analysis. Evol Bioinform Online.

[11] Fofana IJ, Silue S, Diarrassouba N, Kadio AA, Sangare A (2013). Comparative analyses of amplified fragment length polymorphism (AFLP) and simple sequence repeat (SSR) in genetic diversity of Teak (*Tectona grandis* Lf). Int J Adv Agric Res.

[12] Gargouri S, Hamza S, Hajlaoui M (2006). AFLP analysis of the genetic variability and population structure of the wheat crown rot fungus *Fusarium pseudograminearum* in Tunisia. Tunis J Plant Prot.

[13] Ghasemkhani M, Holefors A, Marttila S, Dalman K, Zborowska A, Rur M, Rees-George J, Nybom H, Everett KR, Scheper RWA, Garkava-Gustavsson L (2016). Real-time PCR for detection and quantification, and histological characterization of *Neonectria ditissima* in apple trees. Trees.

[14] Krüger J (1974). Zur Genetik von *Nectria galligena* Bres. J Phytopathol.

[15] Lenormand T (2002). Gene flow and the limits to natural selection. Trends Ecol Evol.

[16] Li L, Wanapu C, Huang X, Huang T, Li Q, Peng Y, Huang G (2011). Comparison of AFLP and SSR for genetic diversity analysis of *Brassica napus* hybrids. J Agric Sci.

[17] Mahoney EM, Milgroom MG, Sinclair WA (1999). Origin, genetic diversity and population structure of *Nectria coccinea* var. *faginata* in North America. Mycologia.

[18] Mantel N (1967). The detection of disease clustering and a generalized regression approach. Cancer Res.

[19] Marra RE, Corwin JA (2009). Isolation and characterization of codominant markers for the perennial canker fungal pathogen *Neonectria ditissima*. Mol Ecol Resour.

[20] McCracken AR, Berrie A, Barbara DJ, Locke T, Cooke LR, Phelps K, Swinburne TR, Brown AE, Ellerker B, Langrell SRH (2003). Relative significance of nursery infections and orchard inoculum in the development and spread of apple canker (*Nectria galligena*) in young orchards. Plant Pathol.

[21] McDonald BA (1997). The population genetics of fungi: tools and techniques. Phytopathology.

[22] McDonald BA, Linde C (2002). Pathogen population genetics, evolutionary potential, and durable resistance. Annu Rev Phytopathol.

[23] McDonald BA, McDermott JM (1993). Population Genetics of Plant Pathogenic Fungi. Bioscience.

[24] Nath VS, Senthil M, Hegde VM, Jeeva ML, Misra RS, Veena SS, Raj M (2013). Genetic diversity of *Phytophthora colocasiae* isolates in India based on AFLP analysis. 3 Biotech.

[25] Plante F, Hamelin RC, Bernier L (2002). A comparative study of genetic diversity of populations of *Nectria galligena* and *N. coccinea* var. *faginata* in North America. Mycol Res.

[26] Pringle A, Taylor JW (2002). The fitness of filamentous fungi. Trends Microbiol.

[27] Seymour FA, Crittenden PD, Dickinson MJ, Paoletti M, Montiel D, Cho L, Dyer PS (2005). Breeding systems in the lichen-forming fungal genus *Cladonia*. Fungal Genet Biol.

[28] Shaner G, Finney RE (1977). Effect of nitrogen-fertilization on expression of slow-mildewing resistance in Knox wheat. Phytopathology.

[29] Sun X, Kang S, Zhang Y, Tan X, Yu Y, He H, Zhang X, Liu Y, Wang S, Sun W (2013). Genetic diversity and population structure of rice pathogen *Ustilaginoidea virens* in China. PLoS One.

[30] Tamura K, Dudley J, Nei M, Kumar S (2007). MEGA4: molecular evolutionary genetics analysis (MEGA) software version 4.0. Mol Biol Evol.

[31] Van Lê A, Gladieux P, Lemaire C, Cornille A, Giraud T, Durel CE, Caffier V, Le Cam B (2012). Evolution of pathogenicity traits in the apple scab fungal pathogen in response to the domestication of its host. Evol Appl.

[32] Walser J, Gugerli F, Holderegger R, Kuonen D, Scheidegger C (2004). Recombination and clonal propagation in different populations of the lichen *Lobaria pulmonaria*. Heredity.

[33] Weber RWS (2014). Biology and control of the apple canker fungus *Neonectria ditissima* (syn. *N. galligena*) from a Northwestern European perspective. Erwerbs-Obstbau.

[34] Weising K, Nybom H, Pfenninger M, Wolff K, Kahl G (2005). DNA fingerprinting in plants: principles, methods, and applications.

[35] Xu X, Harvey N, Roberts A, Barbara D (2013). Population variation of apple scab (*Venturia inaequalis*) within mixed orchards in the UK. Eur J Plant Pathol.

[36] Yeh FC, Yang R, Boyle TB, Ye Z, Mao JX (1997). POPGENE, the user-friendly shareware for population genetic analysis.

